# Composition analysis and prebiotics properties of polysaccharides extracted from *Lepista sordida* submerged cultivation mycelium

**DOI:** 10.3389/fmicb.2022.1077322

**Published:** 2023-01-12

**Authors:** Lanying Wang, Junhui Lian, Qinhua Zheng, Liang Wang, Yanzhen Wang, Dongsheng Yang

**Affiliations:** College of Pharmacy and Food Science, Zhuhai College of Science and Technology, Zhuhai, China

**Keywords:** *Lepista sordida* polysaccharides, ultrasonic-micro wave synergy extraction method, monosaccharide composition, antioxidant, prebiotics

## Abstract

In this paper, Lepista sordida polysaccharides (LSP) were separated from *Lepista sordida* (*L. sordida*) mainly using the Ultrasonic-Micro Wave Synergy Extraction (UMSE) method and purified by graded alcohol precipitation. Three polysaccharide components: 40%-LSP-UMSE, 60%-LSP-UMSE, and 80%-LSP-UMSE were obtained and further analyzed the physicochemical properties, structural characteristics, and antioxidant activity. And the effects on the proliferation of *Lactobacillus casei* of three polysaccharide components were studied. The characteristic absorption peaks and the β-glycosidic bond of three polysaccharide components were the direct expression at UV 200 nm using UV and FT-IR spectroscopy. The three polysaccharide components were mainly composed of glucose, mannose, galactose, and ribose using high-performance liquid chromatography (HPLC) analysis. The antioxidant activity study revealed that the polysaccharides obtained by the UMSE method had better antioxidant activity compared to the traditional “Hot Water Extraction (HWE)” method. In addition, the polysaccharide components promoted the proliferation of *L. casei* to some extent. 40%-LSP-UMSE, 80%-LSP-UMSE as the carbon source had better acid production than the control inulin. Three LSP-UMSE used as a carbon source compared with glucose for culturing *L. casei* could significantly improve its tolerance to bile salts. Results are helpful to develop the bioactive polysaccharides from *Lepista sordida* and beneficial to develop a unique health and functional product in the future.

## 1. Introduction

*Lepista sordida* (Schum. Fr.) Sing is a basidiomycete fungus of the family Trichoderma spp. It is widely distributed throughout China and has attracted much attention due to its high nutritional content and delicious taste. So far, a variety of chemical components such as amino acids, terpene lactones, polysaccharides, and trace elements have been isolated from this fungus, which is a high-value edible fungus. In recent years, *L. sordida* polysaccharide (LSP) developed from *L. sordida* has been considered as a natural phytoconjugate and is quite popular. Several reports have shown that LSP has antioxidant, anticancer, antimicrobial, and probiotic-promoting effects (Xenia et al., 1996; Qiang et al., 2012; Zhong et al., 2013; Miao et al., 2013a; Xu et al., 2021).

Polysaccharide is an important bioactive compound, which is widely found in nature. As an important source of polysaccharides, polysaccharides from edible fungi are not only rich in variety but also have many pharmacological activities on the human body, such as anti-tumor (Miao et al., 2013b), immune regulation (Hongyan et al., 2020; Zhang et al., 2022), Anti-bacteria (Abdullah et al., 2010), antioxidant (Zhong et al., 2013) and anti-inflammatory (Zhenzhen et al., 2018) effects (Kozarski et al., 2012; Zhong et al., 2013; Zhenzhen et al., 2018; Jixian et al., 2018; Jiao et al., 2019; Hongyan et al., 2020; Goldman et al., 2021; Wang et al., 2021). According to Wang et al. (2015) it is known that the optimum temperature, initial pH, rotational speed, medium volume (ratio of medium volume to bottle volume), and inoculum volume for mycelial growth affect the maximum content of bioactive polysaccharides. Even the same raw material with different extraction methods will have different activities of polysaccharides. Ultrasonic - microwave synergistic extraction method makes full use of the high-energy effect of microwave and ultrasonic vibration of cavitation, open microwave and ultrasonic oscillation combined with a new extraction technology, this technology overcomes the shortcomings when using microwave or ultrasonic alone, in a low-temperature atmospheric pressure environment, to achieve rapid, reliable and efficient extraction of the sample. Compared with the traditional extraction methods, the advantages of ultrasonic-microwave synergistic extraction are the extremely short extraction time of polysaccharides, low temperature, and low cost of subsequent processing, etc. There are few reports in the literature on microwave-ultrasonic synergistic extraction of polysaccharides, so this paper uses microwave-ultrasonic synergistic extraction of polysaccharides from *L. sordida*.

Prebiotics are undigested or non-digestible food components that are degraded by the host’s intestinal flora to produce secondary metabolites that have positive effects on the host’s health, such as modulating immunity, improving the intestinal micro-ecosystem, promoting mineral absorption and regulating the body’s metabolism (Keulers et al., 2022; Yang et al., 2022; Yilmaz et al., 2022). Prebiotics are mainly functional oligosaccharides, microalgae (Spirulina, plant extracts, soil extracts, etc.) and polysaccharides. Polysaccharides from edible and medicinal mushrooms have prebiotic effects similar to those of functional oligosaccharides. Some polysaccharides of edible and medicinal mushrooms can be utilized by probiotics in the digestive tract and produce short-chain fatty acids (SCFAs; Yuntao et al., 2021). At the same time, Bacillus polysaccharides as prebiotics have good effects on improving the number and structure of intestinal flora and are also important for improving the intestinal micro-ecosystem (Sidharth et al., 2019). The intestinal micro-ecosystem consists of intestinal tissues, cells, intestinal flora, and metabolites. More than 98% of the intestinal flora are Firmicutes and Bacteroidetes, which are the dominant flora. Only when the structure and quantity of intestinal flora are in dynamic balance in the host body can the organism be maintained to participate in normal physiological activities such as digestion and absorption, energy metabolism, and immune response (Dawood et al., 2021). This balance is disturbed when the number or structure of intestinal flora changes, causing cardiovascular disease, gastrointestinal cancers, diabetes, obesity, autoimmune diseases and inflammatory bowel disease, as well as other diseases that endanger the health of the organism (Yang et al., 2021; Sun et al., 2022; Wu et al., 2022). For example, alterations in the ratio of Bacteroides to Firmicutes have been associated with diseases such as cancer, obesity, and diabetes (Liuming et al., 2020). Probiotics are non-pathogenic microorganisms, mainly of human origin, which, when used in appropriate amounts, are beneficial to the health of the host and can prevent or improve certain diseases (Lee et al., 2002; Fric, 2007; Rabia and Nagendra, 2011). *Lactobacillus casei* (*L. casei*) is a common probiotic in the human gastrointestinal tract and one of the most widely used probiotics. Therefore, it is representative and applicable to study the effect of polysaccharides on *L. casei*.

In this study, LPS was extracted by the UMSE method, and its antioxidant activity was compared with that obtained by hot water extraction method, and finally by using 40%-LSP-UMSE, 60%-LSP-UMSE, and 80%-LSP-UMSE as carbon sources to observe their effects on *L. casei*. In order to provide a scientific basis for the development of LPS and *L. casei* combinations and a theoretical reference for future basic research on LPS probiotics.

## 2. Materials and methods

### 2.1. Materials and chemicals

Concentrated sulfuric acid, phenol, and anhydrous ethanol (C_2_H_5_OH, AR) were purchased from Sinopharm Chemical Reagent Co., Ltd. Glucose standards, glucose (AR), Coomassie Brilliant Blue, bovine serum albumen (BSA), D(+) lacturonic acid, M - hydroxybenzidine, monosaccharide standard, PMP derivative reagent, KBr (AR), salicylicacid (AR), Tris (AR), bile salts, potassium hexacyanoferrate (III; K_3_[Fe(CN)_6_]; AR), purchased from Shanghai YuanYe Biotechnology Co., Ltd. Acetonitrile was purchased from Thermo Fisher Scientific Co., Ltd. (China). DNS (AR) was purchased from Guangzhou Testing Technology Co., Ltd. DPPH (AR) was purchased from Aladdin. Ascorbic acid (AR) Tianjin Kemiou Chemical Reagent Co. Ltd. FeSO_4_•7H_2_O (AR), 30% H_2_O_2_ (AR), K_2_(SO_4_)_2_ (AR), Trichloroacetic acid (AR), FeCl_3_ (AR) Xilong Chemical Co. Ltd. MRS medium and MRS medium without glucose Guangdong Haibo Biological Technology Co., Ltd. Inulin China Eclipse Moon Health Technology Co., Ltd. *Lactobacillus casei* was purchased from Guangdong Province General Microbiological Culture Collection Center. *Escherichia coli* (*E. coli*) and *Staphylococcus aureus* (*S. aureus*) were purchased from the Guangdong Huankai Microbiology Technology co., Ltd. MH medium and NB medium were purchased from Haibo Biotechnology Co., Ltd. Dye resazurin Shanghai Haibo Biotechnology Co., Ltd.

### 2.2. Microorganism, inoculum preparation, and flask cultures

The test strain JZ01 of *L. sordida* was provided by the microbiology laboratory of Zhuhai College of Jilin University, and was cultured with PDA solid medium for 10 days continuously at 25°C. The liquid strain was prepared after 2 generations of recovery, i.e., mass inoculation with 3% inoculum, and incubated at 28°C, 150 rpm/min for 7 days. After centrifugation to remove the fermentation broth, the fermentation mycelium was rinsed with distilled water and a color development test was carried out by phenol sulfuric acid method until no color was shown and the total sugar content of wastewater was close to 0 mg/ml. After centrifugation to remove water, the mycelium was freeze-dried and stored.

### 2.3. Preparation, separation, and purification of LSP

#### 2.3.1. Preparation of LSP

The *L. sordida* mycelium was crushed and sieved through 60 mesh, and the extraction time was 20 min with distilled water as the solvent, the material-liquid ratio of 1:20 g/ml, microwave power of 100 w, and ultrasonic power of 100 w. The extract was centrifuged and the supernatant was concentrated under reduced pressure and then graded for alcoholic sedimentation with vigorous stirring to obtain polysaccharide extracts with ethanol concentrations of 40, 60, and 80%, respectively, overnight at 4°C. The precipitate was collected by centrifugation and dried to obtain crude LPS.

The total sugar content of crude polysaccharides was determined by the phenol-sulfuric acid method (Qiang et al., 2012), and the yield of crude LSP was calculated by the following formula:


Yield(%)=weight of crudeLSP(g)/weight ofLPpowder(g)×100

#### 2.3.2. Hot water extraction method

The optimal *hot water* extraction parameters were as follows: extraction time of 2.86 h, temperature of 92°C, and liquid to solid ratio of 72:1. After the extraction, the extracts were centrifuged, filtered and then concentrated to 1/4 volume and finally cooled to 37°C. Ethanol graded precipitation and subsequent steps are performed in the same way as in “2.3.1. Preparation of LSP”.

### 2.4. Determination of physicochemical properties of LPS

#### 2.4.1. Reducing sugar determination

The content of acidic polysaccharides in LP was determined by the m-hydroxybiphenyl method with D-galacturonic acid as the control (Liu et al., 2019). The content of reducing sugars in LP was determined by the DNS method using glucose as standard (Zhang et al., 2019; Liu et al., 2019). The crude LSP protein content was determined by the Kormas Brilliant Blue method using Bradford bovine serum albumin (BSA) as the standard (Bradford, 1976).

#### 2.4.2. Molecular weight determination

The molecular weights of the three groups of polysaccharides (Mw) were determined by high performance liquid chromatography (HPLC) using Agilent 1,100 HPLC and gel chromatography column TSK-G3000. The samples were dissolved in distilled water and filtered through a 0.45um polyethersulfone membrane, respectively. The column was run at 30°C with a loading volume of 20ul at a flow rate of 0.8 ml/min and isocratic elution with 50 mM ammonium acetate solution, connected to an ELSD detector (55°C) and pass N_2_ (gas flow rate 2.0/mL) for dextran standards of different molecular weights.

#### 2.4.3. Monosaccharide composition analysis

##### 2.4.3.1. Hydrolysis of polysaccharides

Weigh 20.0 mg of polysaccharide sample and add 10 ml of 2 mol/L TFA, seal the tube with N_2_ (10 L/min, 1 min), and hydrolyze at 100°C for 2 h. After hydrolysis, remove the TFA with methanol, blow dry the methanol with N_2_, and repeat blow dry twice to remove the TFA; add an appropriate amount of NaOH solution to dissolve the substrate.

##### 2.4.3.2. PMP derivative process

A small amount of mixed monosaccharide standard solution or polysaccharide hydrolysis solution was taken and added to an equal volume of PMP methanol solution for 2 h at 70°C; the reaction was cooled to room temperature and 400 μl of 0.3 mol/L HCl was added to neutralize (pH 6 ~ 7); the mixture was diluted with water, then an equal volume of chloroform was added to extract, and the chloroform phase was discarded, and the extraction was repeated twice and then filtered through 0.45 μm aqueous microporous membrane.

##### 2.4.3.3. HPLC detection conditions

The monosaccharide composition of the polysaccharide components was determined by high-performance liquid chromatography (HPLC) using Agilent 1,100 HPLC and C18 column (250 mm*4.6 mm, 5 μm). Mobile phase A was 90 mmol/L sodium phosphate buffer (pH = 7.8) and mobile phase B was acetonitrile (AN). The injection volume was 10 μl, the flow rate was 1 ml/min, the column temperature was 30°C and the detection was monitored at a wavelength of 250 nm (Yi et al., 2011).

#### 2.4.4. FT-IR and UV spectroscopic analyses

##### 2.4.4.1. UV–visible spectrum analysis

The LSP (40%-LSP-UMSE, 60%-LSP-UMSE, 80%-LSP-UMSE) was scanned by ultraviolet in the range of 200–400 nm using a UV–visible spectrum (Shimadzu, Japan) and LSP concentration was 0.05 mg/ml (Bradford, 1976; Yi et al., 2011).

##### 2.4.4.2. Fourier transform infrared spectrometer analysis

LSP was analyzed using IR Prestige-21 (Shimadzu, Japan). LSP (2 mg) was mixed with potassium bromide (KBr) powder (1:100), ground, and tableted, and then the infrared spectrum was scanned in the wave number range of 400 cm^−1^ ~ 5,000 cm^−1^ (Fujia et al., 2017; Jixian et al., 2018).

### 2.5. Antioxidant activity of mycelial polysaccharides *in vitro*

#### 2.5.1. DPPH radical scavenging activity

The reaction mixture that contained different concentrations of LPS (1 ml, 0.01-8 mg/ml) and DPPH anhydrous ethanol solution (1 ml, 0.1 mmol) was mixed with sufficient shaking and incubated at room temperature for 30 min in the dark. Then, the mixture was centrifuged (8,000 rpm for 5 min), and the absorbance at 517 nm was measured (Jingjing et al., 2012). Each group was operated in parallel three times. Lower absorbance of the reaction mixture indicated higher free-radical-scavenging activity. The anhydrous ethanol and the sample solution were the controls, the distilled water instead of the sample solution was used as the blank. The same concentration of ascorbic acid was used as a positive control. The result was calculated as follows:


$$\mathrm{Scavenging ability}\;\left(\%\right)=\left[1-\left(A_{i}-A_{j}\right)/A_{0}\right]\times 100$$

where A_j_ was the absorbance value of the blank group, A_0_ was the absorbance value of the control group, A_i_ was the absorbance value of the sample group.

#### 2.5.2. Hydroxyl radical scavenging activity

The hydroxyl radical-scavenging activity was determined by the salicylic acid method (Liuming et al., 2020). Different concentrations of polysaccharide extracts, 0.50 ml of FeSO_4_ solution (9 mmol/L), and 0.50 ml of salicylic acid ethanol (9 mmol/L) solution were mixed. Then, 0.25 ml of H_2_O_2_ solution (8.8 mmol/L) was added and the mixture was incubated at 37°C for 30 min. After the mixture was centrifuged (8,000 rpm for 5 min), The absorbance was determined at 510 nm. The calculation of Hydroxyl radical-scavenging activity is the same as that of DPPḤ radical.

#### 2.5.3. ABTS radical scavenging activity

The ABTS radical scavenging activity of the extracted polysaccharides was determined using a standard method (Tan et al., 2020), slightly modified as follows. ABTS ^+^ solution was made by mixing potassium persulfate (2.45 mM) with ABTS (7.0 mM) for 12 h. The prepared ABTS solution was diluted 40–45 times with phosphate buffer (pH 7.4), to make its absorbance 0.70 ± 0.02 at 734 nm, thereafter, 1.50 ml of ABTS ^+^ was mixed with 0.50 ml of the sample at different concentrations. The mixture was shaken with a vortex and incubated for 6 min before the absorbance is recorded at 734 nm. V_C_ was used as a positive control. The formula used for calculation is the same as for the DPPḤ radical.

#### 2.5.4. Ferric-reducing antioxidant power

The ferric-reducing power of the extracted polysaccharides was evaluated using a previously reported method (Yin et al., 2021) with minor modifications. The polysaccharides were dissolved in distilled water at different concentrations (0.05, 0.1, 0.25, 0.5, 1.0, 2.0, 4.0, 6.0, and 8.0 mg/ml) and 0.50 ml polysaccharide solution was mixed with 1.0 ml of sodium phosphate buffer (0.2 M, pH 6.6) and 1.0 ml of 10% potassium ferricyanide. The mixture was incubated at 50°C for 30 min before adding 1.0 ml trichloroacetic acid (10% w/v). After centrifugation at 4,000 × *g* for 15 min, the supernatant was taken out and mixed with equal volumes of deionized water and 1/5 equal volumes of FeCl_3_. After 30 min, absorbance at 700 nm was recorded. Ascorbic acid served as a positive control. The ferric-reducing power of the polysaccharides was calculated using Eq.


$$\mathrm{Reducing power}=A_{1}-A_{2}$$

where A_1_ is the absorbance of the polysaccharides and A_2_ is the absorbance of the distilled water, which replaced the FeCl_3_ solution.

### 2.6. Prebiotic of LSP-UMSE on *Lactobacillus casei*

*Lactobacillus casei*, a Lactobacillus that contains relatively high levels of β-glucosidase and glycotransferase, can enhance its activity by breaking down prebiotics into smaller units for use (Rabia and Nagendra, 2011).

#### 2.6.1. LSP safety assessment

##### 2.6.1.1. MICs determination

The assay was done according to the modified method of Abdullah et al. (2010) *Staphylococcus aureus* (*S. aureus*), and *Escherichia coli* (*E. coli*) were cultivated in a liquid medium for reactivation. The suspension was transferred into a fresh, sterile MH medium, resulting in a final concentration of (1 ~ 2) × 10^6^ CFU/ml.

The polysaccharide sample was weighed 200 mg and dissolved in 5 ml of water with the aid of ultrasonic cleaner to reach the concentration of 40 mg/ml. The polysaccharide concentration was diluted twice in a 96-well plate according to the two-fold dilution method, and the dilution medium was sterile NB medium. 100 μl of each was inoculated with a bacterial solution and incubated for 22 h at 37°C. After mixing, the wells were incubated at 37°C for 2 h. When the wells turned pink, the bacteria were proliferating, and when the wells showed light blue or dark blue, the bacteria were inhibited from growing. All the above operations were done under aseptic conditions.

##### 2.6.1.2. Effect of LPS on the growth of *Lactobacillus casei in vitro*

*Lactobacillus casei* was diluted to a final concentration of (1 ~ 2) × 10^6^ CFU/ml using a McClatchy turbidimeter and sterile sugar-free MRS medium. 20, 15, 10, 5, 2.5, 1.25, 0.625, 0.315, 0.157, 0 mg/ml of polysaccharide MRS medium was prepared using sugar-free MRS, and each well was inoculated with 4% of the diluted suspension and mixed well. The absorbance at 600 nm was measured at 0 h and 22 h. After 22 h, 40 μl of 0.2 mg/ml of the solution was added to each well and incubated for 2 h. The OD_600_ and the discoloration of the wells were used to determine whether the 40%-LSP-UMSE, 60%-LSP-UMSE, and 80%-LSP-UMSE had the ability to produce the desired effect. 40%-LSP-UMSE, 60%-LSP-UMSE, and 80%-LSP-UMSE were used to determine whether they were bacteriostatic. All operations were performed under aseptic conditions.

#### 2.6.2. Prebiotic activity of LSP

Referring to the method (Mohd et al., 2017) and modified, 40%-LSP-UMSE, 60%-LSP-UMSE, and 80%-LSP-UMSE, inulin, and glucose were used as carbon sources to prepare complex medium, and the polysaccharide mass concentrations 0, 0.1, 0.2, 0.3, 0.4, 0.5, 1.0, 1.5, and 2.0%, respectively. After sterilization and cooling to room temperature, the insoluble material was removed by centrifugation. Inoculated with 4% 1.5 × 10^8^ CFU/ml *L. casei* suspension and incubated at 37°C for 72 h. OD_600_ was measured at 0, 4, 8, 12, 16, 20, 24, 28, 36, 48, and 72 h. The positive control was inulin and glucose. Each sample was operated on three times in parallel.

#### 2.6.3. Effect of polysaccharide carbon source on probiotics

The method was performed based on a published assay with some modifications (Xu et al., 2022). The 0.5% complex polysaccharide MRS medium with a glucose-polysaccharide ratio of 9:1 was prepared using sugar-free MRS medium, 10 ml each, and sterilized for use. After inoculation with 4% 1.5 × 10^8^ CFU/ml *L. casei* suspension, the culture was incubated at 37°C for 72 h. The OD_600_ was measured at 0, 4, 8, 12, 16, 20, 24, 28, 36, 48 and 72 h. The inulin complex polysaccharide group was used as a positive control for the prebiotic activity of LSP. Whether each 0.5% complex polysaccharide MRS medium had the effect of promoting acid production was also determined, and the acidity value was used as an evaluation index. The acidity values were determined using potentiometric titration combined with a phenolphthalein indicator. The formula for calculating the acidity value is as follows:


$$\mathrm{Acidity values}\;\left({}^{0}T\right)=C_{1}\;\left(V_{1}-V_{0}\right)\times 100/\mathrm{mL}\times 0.1$$

where C_1_ is the concentration of sodium hydroxide per liter used for calibration; V_1_ is the volume of sodium hydroxide milliliter consumed by fermentation broth; V_0_ is the volume of sodium hydroxide consumed in distilled water; 100 is for every 100 ml of fermentation liquid; M_1_ is the actual sample volume.

#### 2.6.4. Optimum culture concentration for 24 h

The inoculum was sterilized, and the insoluble material was removed by centrifugation under aseptic conditions and set aside. The inoculation amount was 4% 1.5 × 10^8^ CFU/ml *L. casei* suspension, and the optical density at 600 nm was measured after 24 h incubation at 37°C. The positive control group was an inulin carbon source, the same as above, and the blank control was a sugar-free MRS medium. Each sample was operated on three times in parallel.

#### 2.6.5. Determination of bile salt tolerance activity

40%-LSP-UMSE, 60%-LSP-UMSE, 80%-LSP-UMSE, and inulin were used as carbon sources to prepare 10 mg/ml polysaccharide MRS medium using sugar-free MRS medium, which was sterilized, centrifuged and discarded insoluble material, and set aside. After inoculation with 4% 1.5 × 10^8^ CFU/ml *L. casei* suspension, the suspension was incubated at 37°C for 24 h and then washed and precipitated by centrifugation to obtain a live bacterial precipitate without fermentation broth and polysaccharide, and the bile salt resistance activity was measured after preparing the live bacterial suspension with the same volume of sterile saline as the original fermentation broth. *L. casei* in the 0.5% glucose group was used as a negative control.

The bile salt was prepared by sterile MRS medium at room temperature to 0.3% bile salt medium, and then the saline suspension was inoculated with 4% inoculum and incubated in a water bath shaker at 37°C at 130 rpm/min for 6 h. The OD_600_ was measured at 0, 1, 2, 3, 4, 5, and 6 h. The 10 mg/ml inulin carbon source group was used as a positive control, and the 0.5% glucose carbon source group was used as a negative control. The group was operated on three times in parallel. The changes in bile salt resistance activity of *L. casei* after polysaccharide culture were analyzed by OD_600_.

### 2.7. Statistical analysis

All samples were analyzed in triplicate and averaged. The obtained data were presented as means of three determinations within significance *p* < 0.05. The Statistics Package for Social Sciences 25 (IBM SPSS Statistics 25) was employed for the regression analysis and optimization. All graphs were created and calculations were performed using Origin 2018 and Graphpad Prism 8, respectively.

## 3. Results and discussion

### 3.1. Analysis of chemical components

The results of the chemical composition analysis of LSP were shown in Table 1.

**Table 1:** Chemical components and monosaccharide composition of LSP.

Fraction	Yield (%)	Total sugar (%)	Reducing sugars (%)	Acidic polysaccharide (%)	Protein (%)
40%-LSP-UMSE	41.02%	41.02%	0.00%	1.35%	5.80%
60%-LSP-UMSE	38.46%	38.46%	0.00%	1.57%	0.70%
80%-LSP-UMSE	5.62%	16.92%	11.30%	0.67%	0%

### 3.2. Analysis of the molecular weights of the LPS

**Figure 1:**
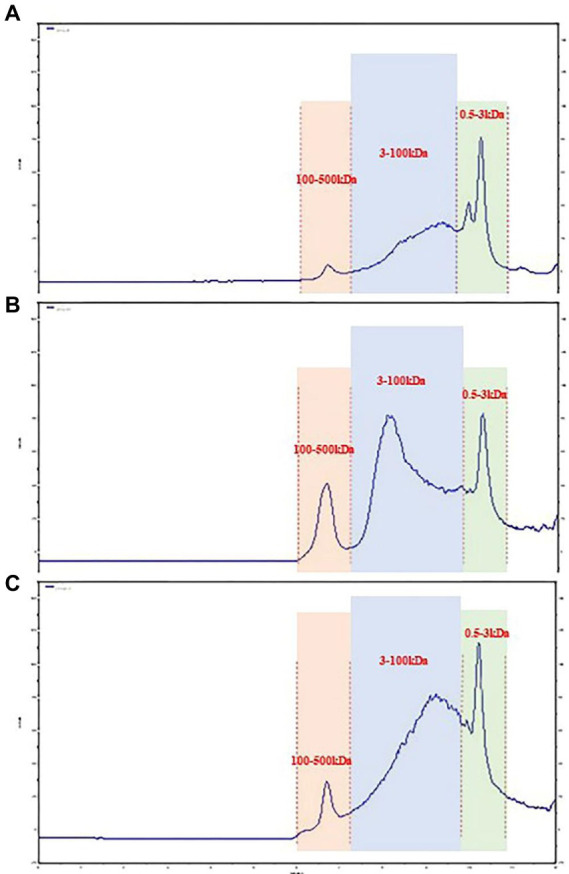
Molecular weight analysis of LPS. **(A)** 40%-LSP-UMSE, **(B)** 60%-LSP-UMSE, **(C)** 80%-LSP-UMSE.

In recent years, size-exclusion chromatography combined with multi-angle laser scattering has been considered as an effective and reliable method to determine the molecular characteristics of biological macromolecules. As shown in Figure 1. The molecular weights of three groups of LPS were determined by HPGPC method. According to the molecular weight standard curve of polysaccharides (logM = −0.6246x + 9.5063Rt, R^2^ = 0.9981) and retention time (RT). the molecular weights of 40%-LSP-UMSE were 1,316–215,236 Da, 60%-LSP-UMSE were 1,243–224,098 Da, and 80%-LSP-UMSE were 1,334–216,169 Da. according to monosaccharide composition analysis, LPS is a heteropolysaccharide, mainly composed of glucose, mannose, ribose and galactose.

### 3.3. Monosaccharide composition analysis

**Figure 2:**
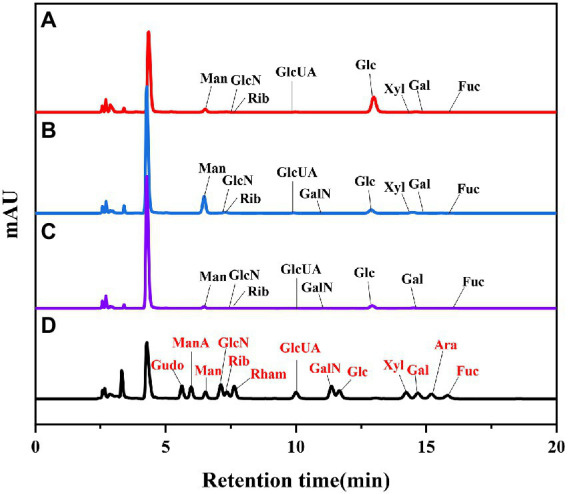
HPLC chromatogram of standard monosaccharide and LSP-UMSE **(A)** standard monosaccharide, **(B)** 40%-LSP-UMSE, **(C)** 60%-LSP-UMSE, **(D)** 80%-LSP-UMSE.

As shown in Figure 2. The biological activity of polysaccharides is usually affected by their monosaccharide compositions. Based on the monosaccharide composition analysis, for LSP, four monosaccharides (glucose, mannose, galactose, ribose) were present in all four polysaccharide preparations, although their molar ratios were different (Table 2). The primary monosaccharides of 40%-LSP-UMSE and 60%-LSP-UMSE were glucose and mannose. The 80%-LSP-UMSE was mainly composed of glucose, mannose, galactose, and ribose.

**Table 2:** Monosaccharide compositions of the LSP.

Fraction	Monosaccharide composition (mg/g)								
	Glc	Man	Gal	Rib	GlcUA	Fuc	Xyl	GlcN	GalN
40%-LSP-UMSE	183.88	22.93	2.59	2.1	1.63	0.61	0.49	0.43	-
60%-LSP-UMSE	41.22	120.4	7.27	8.21	2.68	2.33	0.8125	0.41	0.103
80%-LSP-UMSE	30.87	11.79	4.59	2.09	0.26	1.23	-	0.61	0.06

### 3.4. UV–visible spectrum FT-IR spectrum of LSP

**Figure 3:**
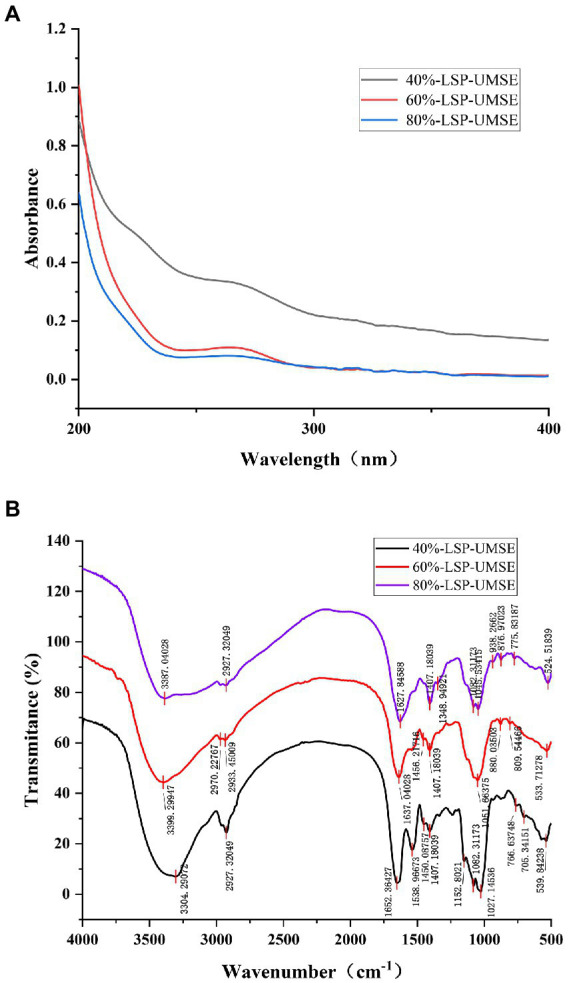
Spectral analysis of 40%-LSP-UMSE, 60%-LSP-UMSE and 80%-LSP-UMSE. **(A)** UV scanning spectra of 40%-LSP-UMSE, 60%-LSP-UMSE and 80%-LSP-UMSE; **(B)** FT-IR spectra of 40%-LSP-UMSE, 60%-LSP-UMSE and 80%-LSP-UMSE.

UV spectrum of LSP-UMSE at 200–400 nm was shown in Figure 3A. There was a strong absorption peak at about 200 nm which was the characteristic absorption peak of polysaccharides (Hao-Bin et al., 2018). 40%-LSP-UMSE and 60%-LSP-UMSE absorption peaks were found at 260 and 280 nm, indicating that the purified polysaccharides may contain nucleic acid and protein (Yongjie et al., 2020).

The FT-IR spectra of the polysaccharides extract isolated from LSP-UMSE (Figure 3B) showed the characteristic strong broad absorption in the range of 3,350–3,405 cm^−1^ corresponding to stretching vibration of O▬H groups, indicating that the LSP-UMSE may contain intramolecular or intermolecular O-H and the absorption peak at 2900 cm^−1^ of LSP-UMSE was derived from the C▬H stretching vibration of CH. The absorption peak near 1,650 cm^−1^ was assigned to the amide group C〓O stretching vibration and the COOH vibration. The peak near 1,420 cm^−1^ was attributed to the C▬H variable angle vibration at the same time, all three components have three absorption peaks at 1020-1150 cm^−1^, indicating that it was a pyran-type polysaccharide. There are also characteristic peaks at 821, 827, and 839 cm^−1^, which indicate the presence of glucose β-terminal heterodyne phase isomerism C-H (Wang et al., 2022), thus it is inferred that the three components have β-glycosidic bond configurations.

### 3.5. *In vitro* antioxidant activity of LSP

#### 3.5.1. DPPH radical scavenging activity

**Figure 4:**
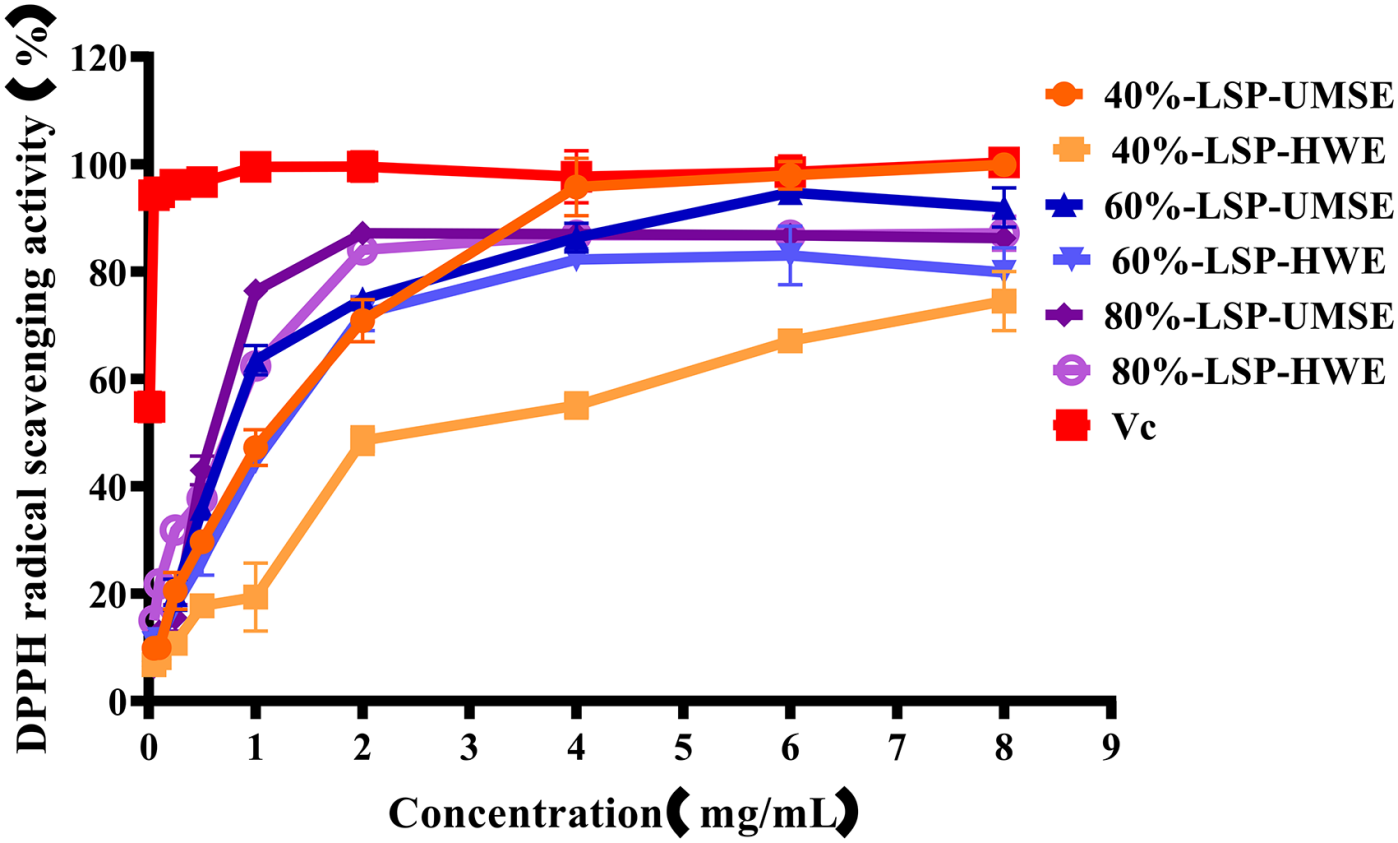
Scavenging ability of LSP-UMSE and LSP-HWE against 2,2-diphenyl-1-picrylhydrazyl (DPPH) radicals. Each value is expressed as mean ± SEM (*n* = 3).

DPPḤ is a very stable organic radical with nitrogen as its center, which has strong absorption at 517 nm in ethanol. It was widely used to evaluate the free-radical scavenging ability of antioxidants (Hao-Bin et al., 2018). As shown in Figure 4, the scavenging activity of the LPS group against DPPH was concentration dependent in the concentration range of 0 ~ 4 mg/ml using V_C_ as a positive control. 40%-LSP-UMSE showed a lower IC_50_ value of 1.125 ± 0.007 mg/ml than 40%-LSP-HWE (IC_50_ of 1.798 ± 0.177 mg/ml). In the range of 0 ~ 8 mg/ml, the scavenging ability of LSP-UMSE for DPPH was significantly better than that of LSP-HWE (*p < 0.05*). At 4 mg/ml, the scavenging rate of 40%-LSP-UMSE was close to that of V_C_. the scavenging rate of DPPH by 60%-LSP-UMSE and 60%-LSP-HWE reached the maximum when the concentration was 2 mg/ml.

#### 3.5.2. Hydroxyl radical scavenging activity

**Figure 5:**
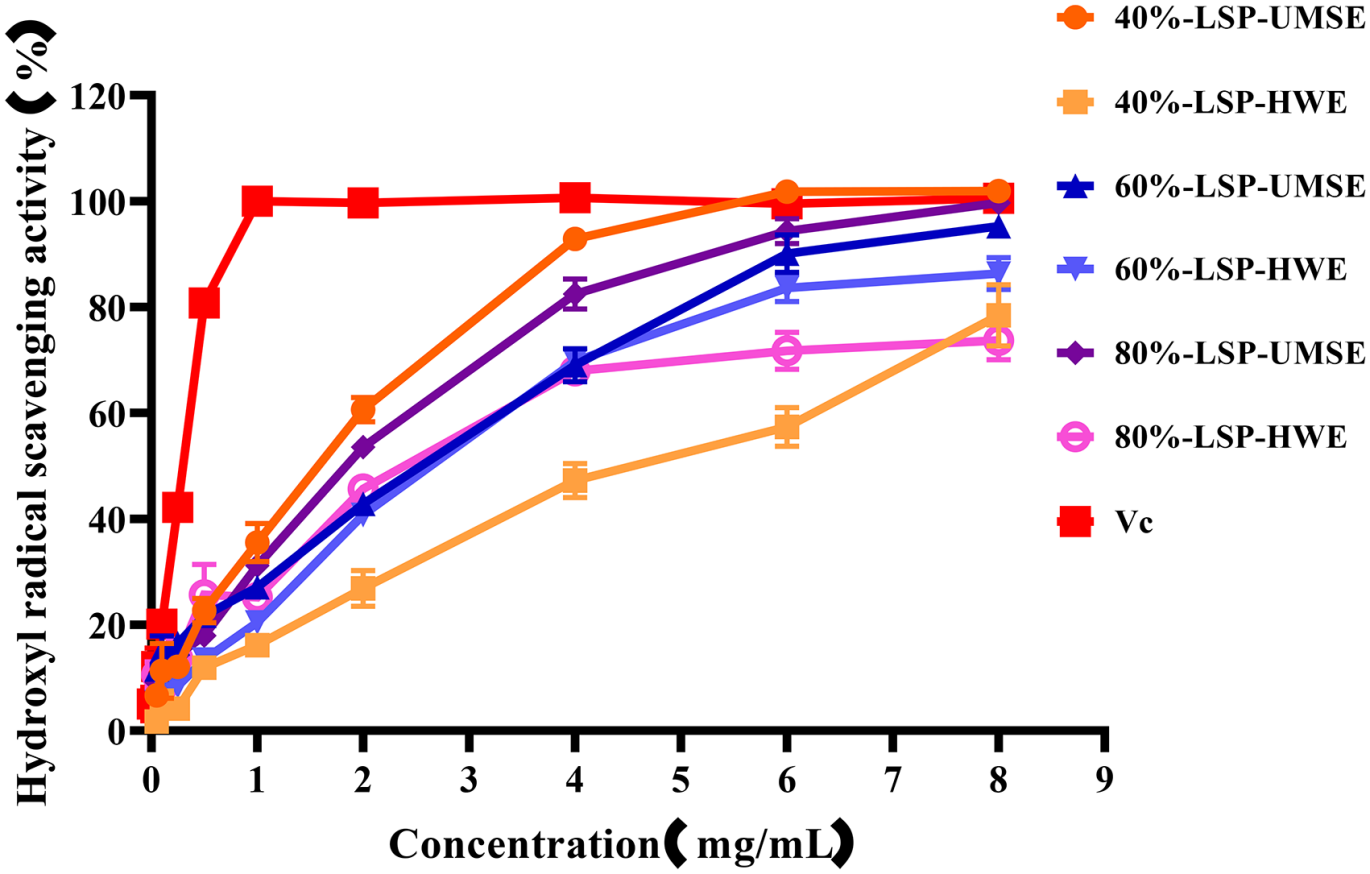
Scavenging ability of LSP-UMSE and LSP-HWE on OH scavenging ability. Each value is expressed as mean ± SEM (*n* = 3).

·OH is dangerous to the organisms through inducing oxidative damage to adjacent biomolecules. Excess hydroxyl radicals are produced during metabolism, leading to oxidation of biological macromolecules such as cell membranes, proteins and enzymes, and leading to cellular oxidation, aging and apoptosis (Hao-Bin et al., 2018). The scavenging ability of LPS on hydroxyl radicals compared with V_C_ used as a positive control is shown in Figure 5. The results showed that 6 each fraction polysaccharide had scavenging ability in the range of 0 ~ 8 mg/ml with concentration dependence. The IC_50_ of 40%-LSP-UMSE, 40%-LSP-HWE, 60%-LSP-UMSE, 60%-LSP-HWE, 80%-LSP-UMSE, 80%-LSP-HWE were 1.125 ± 0.007 mg/ml, 1.798 ± 0.177 mg/ml, 0.759 ± 0.121 mg/ml, 1.013 ± 0.029 mg/ml, 0.532 ± 0.004 mg/ml, and 0.672 ± 0.057 mg/ml were lower than those of *L. sordida* polysaccharides extracted by Shuping et al. (2020); IC_50_ of 4.472 ± 0.745 mg/ml and 4.333 ± 0.298 mg/ml, respectively). The clearance of both 40%-LSP-UMSE and 80%-LSP-UMSE reached a maximum of 100% when the concentration reached 8 mg/ml, which was consistent with the V_C_ clearance. The results indicated that LSP-UMSE had significant scavenging ability for hydroxyl radicals.

#### 3.5.3. ABTS radical scavenging activity

**Figure 6:**
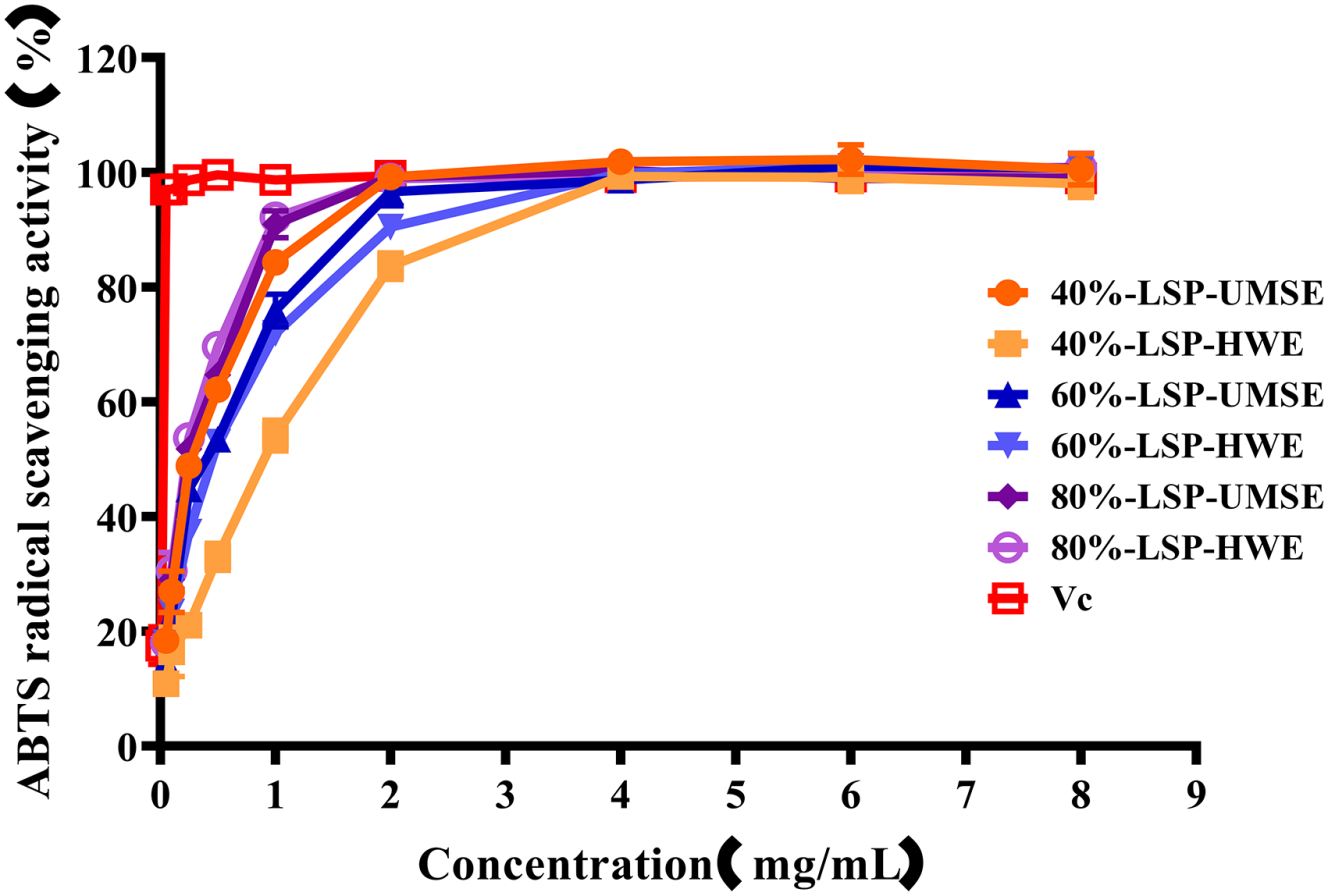
Scavenging ability of LSP-UMSE and LSP-HWE against ABTS radicals scavenging ability. Each value is expressed as mean ± SEM (*n* = 3).

The ABTS cation radical is generated by the oxidation of ABTS with potassium persulfate. When the antioxidant is added, the radical is converted to the non-radical form (Lu et al., 2016). The dependence of solubility at a given concentration can be seen in Figure 6. Compared with V_C_ as a positive control, all the six polysaccharides had a strong ability to scavenge ABTS radicals, and the scavenging ability was enhanced with increasing concentration and then basically stabilized, and in this way the maximum scavenging rate was reached after 2 mg/ml. The IC_50_ of 40%-LSP-HWE was 1.035 ± 0.066 mg/ml lower than the lowest IC_50_ reported in the literature (Shuping et al., 2020; Xu et al., 2021), while there was no significant difference in the scavenging rate of ABTS radicals between LSP-UMSE and LSP-HWE.

#### 3.5.4. Ferric-reducing antioxidant power

**Figure 7:**
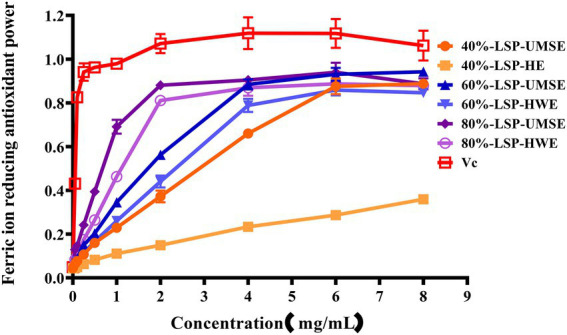
LSP-UMSE and LSP-HWE for ferric ion chelating activity. Each value is expressed as mean ± SEM (*n* = 3).

The ferric-reducing power of the compounds was correlated with the potential antioxidant activity. To measure the reducing power of the six compounds, the ability to transform Fe^3+^ into Fe^2+^ was investigated (Lu et al., 2016). The reducing power of the six compounds is shown in Figure 7, which revealed LSP had a concentration-dependent manner and expressed noticeable reducing power, but it was weaker than V_C_ as a positive control. 60%-LSP-UMSE, 60%-LSP-HWE, 80%-LSP-UMSE, and 80%-LSP-HWE were relatively close in total reducing power at 4 mg/ml. The total reducing power of 60%-LSP-UMSE, 60%-LSP-HWE, 80%-LSP-UMSE, and 80%-LSP-HWE and 40%-LSP-UMSE reached the maximum at a concentration of 6 mg/ml, but was still lower than that of V_C_. In general, the reducing power of LPS in this study was the best compared to that of *L. sordida* polysaccharides reported in the literature (Shuping et al., 2020).

### 3.6. Prebiotic activity of LSP

#### 3.6.1. Effects of LSP on the growth of harmful bacteria

The results were shown in Table 3, within 0 to 20 mg/ml concentration LSP did not show any antibacterial effects on *Staphylococcus aureus* and *Escherichia coli*. The prebiotic rule requires specific activation of probiotic bacteria while inhibiting the growth of pathogenic bacteria. In this study, different concentrations of LPS induced the growth of *S. aureus* and *E. coli*, rather than inhibiting their growth. This result is in consistent with the study of Dawood et al. (2021). However, if it is to be applied to nutraceuticals and food products, subsequent experiments will consider determining its *in vivo* safety and whether it has an indirect role in modulating probiotic activity.

**Table 3:** Effect of LPS on *S. aureus* and *E. coli.*

Concentration (%)	Concentration (mg/ml)										Gentamicin	negative control
	2.00	1.50	1.00	0.50	0.25	0.13	0.07	0.03	0.02	0		
*S. aureus*												
40%-LSP-UMSE	−	−	−	−	−	−	−	−	−	−	++	+
60%-LSP-UMSE	−	−	−	−	−	−	−	−	−	−	++	+
80%-LSP-UMSE	−	−	−	−	−	−	−	−	−	−	++	+
*E. coli*												
40%-LSP-UMSE	−	−	−	−	−	−	−	−	−	−	++	+
60%-LSP-UMSE	−	−	−	−	−	−	−	−	−	−	++	+
80%-LSP-UMSE	−	−	−	−	−	−	−	−	−	−	++	+

“++” The dye resazurin is dark blue, indicating that the concentration has strong bacteriostatic effect; “+” The dye resazurin turned blue, indicating that the concentration began to have bacteriostatic effect; “-” The dye resazurin turned pink, indicating no bacteriostasis.

#### 3.6.2. Effect of LPS on the proliferation of *Lactobacillus casei in vitro*

The OD_600_ difference was obtained based on the color development of dye resazurin in Table 4. The addition of LSP in the concentration range of 0–20 mg/ml had no bacteriostatic effect on *L. casei* and also showed an osmotic bacteriostatic effect, indicating that LSP in the concentration range had no inhibitory effect on the growth of *L. casei* and had a good growth promotion effect.

**Table 4:** *In vitro* safety of LSP against *L. casei* - dye resazurin.

Concentration (%)	2.00	1.50	1.00	0.50	0.25	0.13	0.07	0.03	0.02	0	Gentamicin	Negative control
40%-LSP-UMSE	−	−	−	−	−	−	−	−	−	−	+	+
60%-LSP-UMSE	−	−	−	−	−	−	−	−	−	−	+	+
80%-LSP-UMSE	−	−	−	−	−	−	−	−	−	−	+	+

“++” The dye resazurin is dark blue, indicating that the concentration has a strong bacteriostatic effect; “+” The dye resazurin turned blue, indicating that the concentration began to have bacteriostatic effect; “-” The dye resazurin turned pink, indicating no bacteriostasis.

#### 3.6.3. The optimum concentration for 24 h culture

**Figure 8:**
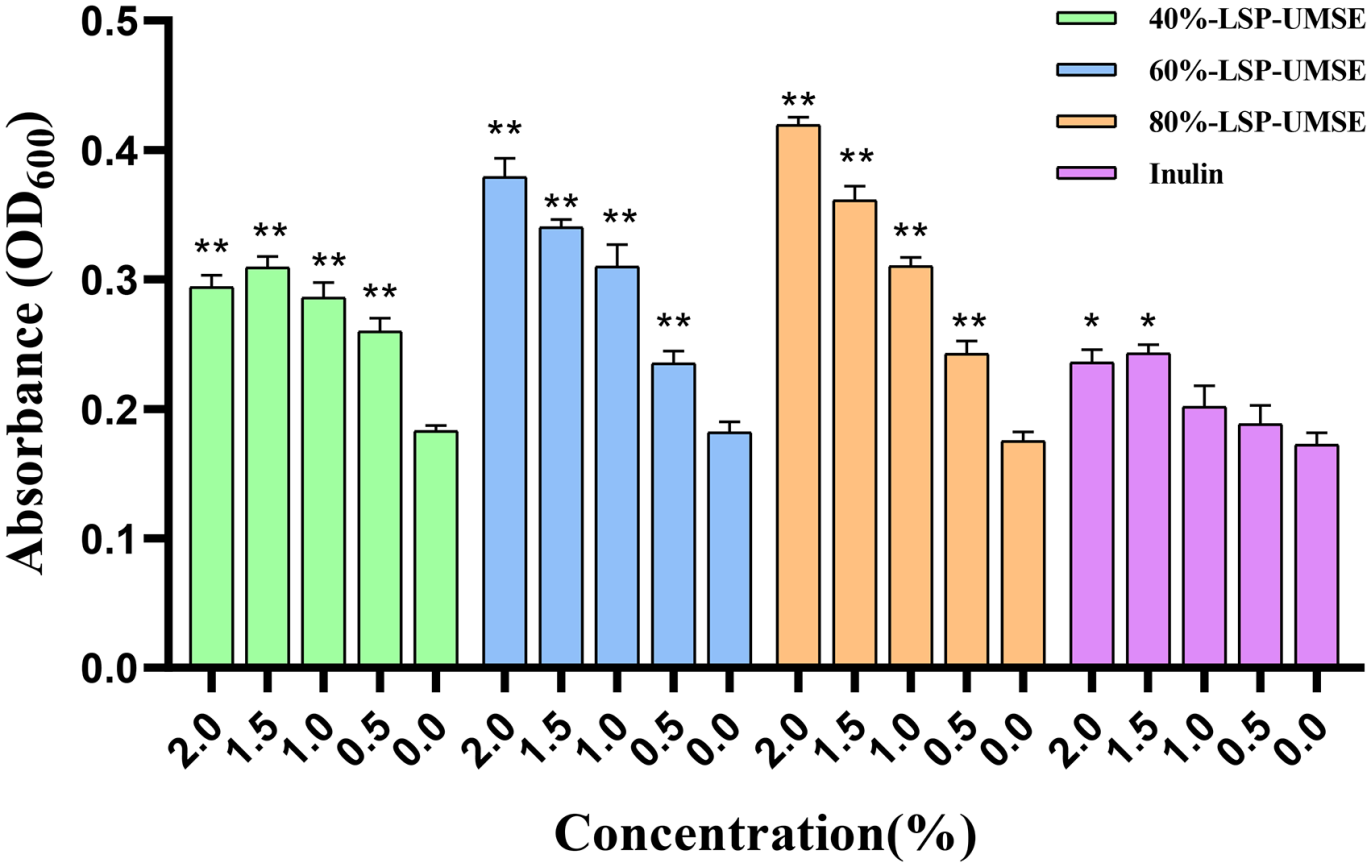
Optimal concentration for 24 h incubation (Note: 0% was used as control, * was significantly *p* < 0.05, ** was extremely significantly *p* < 0.01).

The OD_600_ of different carbon source content media after 24 h incubation is shown in Figure 8. The OD_600_ of the four polysaccharide fractions, 40%-LSP-UMSE, 60%-LSP-UMSE, 80%-LSP-UMSE, and inulin, were all significantly higher than that of the sugar-free media, indicating that *L. casei* was able to utilize all four polysaccharides. At the end of 24 h of incubation, the OD_600_ values in the fermentation medium with LPS as the carbon source increased with the increase of polysaccharide substitution compared with the medium with inulin as the carbon source, in which 80%-LSP-UMSE and 60%-LSP-UMSE had the best proliferation effect with 2% content, and the OD_600_ values increased by 60.59 and 77.65%, respectively, indicating that the addition of LPS promoted the proliferation of *L. casei*. The addition of LPS promoted the proliferation of *L. casei*. In contrast, the OD_600_ values of the 1.5% group at the end of 24 h incubation with 40%-LSP-UMSE decreased with increasing substitution content compared to the OD_600_ values with 2.0% content, indicating that 40%-LSP-UMSE did not fully replace the increased turbidity of the fermentation broth by glucose.

#### 3.6.4. Growth curves of *Lactobacillus casei* cultured in different carbon sources for 72 h

**Figure 9:**
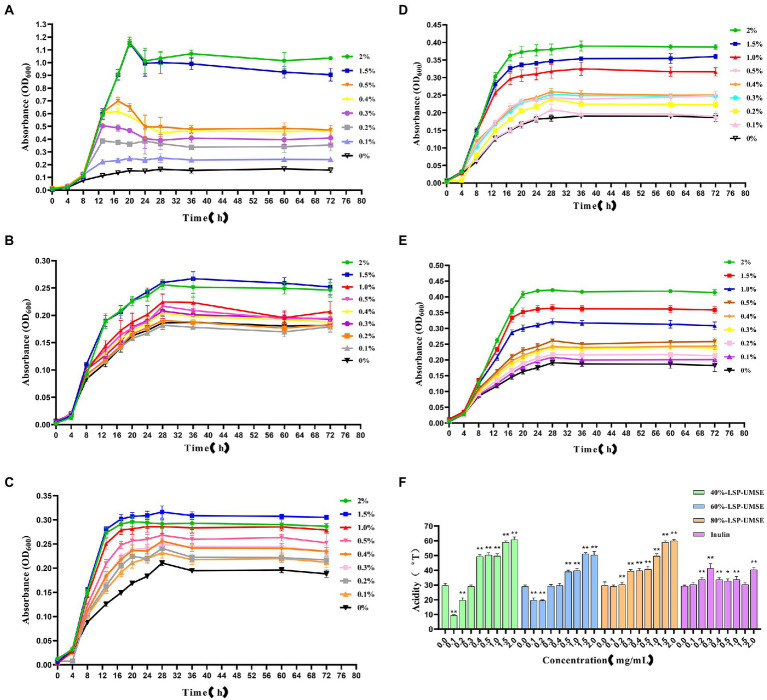
Growth curves of *L. casei* cultured at different carbon source concentrations and acidity values after 24 h incubation at different carbon source concentrations. **(A)** Glucose carbon source; **(B)** inulin carbon source; **(C)** 40%-LSP-UMSE carbon source; **(D)** 60%-LSP-UMSE carbon source; **(E)** 80%-LSP-UMSE carbon source; **(F)** acidity comparison of different carbon source concentrations at 24 h (Note: 0 mg/ml was used as control, * was significantly *p* < 0.05, ** was extremely significantly *p* < 0.01).

As shown in Figure 9A, the 72-h growth curve of the glucose group showed that the growth was delayed from 0 to 4 h, accelerated from 4 to 8 h, and logarithmic from 8 to 18 h. The growth rate of each concentration began to slow down or even decay after 18 to 20 h. The glucose carbon sources in the range of 0.1 to 2.0% at 2.0, 1.5, and 1.0% were relatively longer than the logarithmic growth period in the range of 0 to 0.5%. There was an extension of the log growth period. When glucose was the carbon source, it would enter the decay period after reaching the maximum growth, and the decay rate was different at different glucose carbon source concentrations, where the decay rate at 0.5% was larger than other concentrations.

As shown in Figure 9B, the 72-h growth curve of the inulin group showed that the logarithmic growth period was prolonged from 0 to 4 h, and the logarithmic growth period was from 4 to 18 h. Most of the growth rate began to slow down after 18–28 h until the stabilization period, and the logarithmic growth period was prolonged with the increase of inulin concentration in the concentration range of 0–2.0%, and the growth rate of logarithmic growth period was also increased. Compared with Figure 8A, although the growth rate of the logarithmic growth period of the inulin carbon source was smaller than that of the glucose carbon source, and the maximum bacterial concentration was smaller during the stabilization period, inulin as a polysaccharide carbon source prolonged the growth period of *L. casei* to a certain extent, and there was no decay period, indicating that inulin at least promoted the bacterial viability of *L. casei*, and the promotion effect of inulin concentration on the growth rate of bacteria was positively correlated.

As shown in Figure 9C, the 72-h growth curve of the 40%-LSP-UMSE group showed that 0–4 h was in the delayed period, 4–28 h was in the growth period, where 4–18 h was in the logarithmic growth period, and the growth rate of the bacteria slowly decreased from 18 to 28 h. The whole growth curve showed a similar prebiotic effect as inulin. Compared with Figure 8B, it was found that the growth rate of the 40%-LSP-UMSE carbon source was greater than that of the inulin carbon source in the logarithmic growth period, and the extension of the logarithmic growth period was not obvious. Overall, the 40%-LSP-UMSE carbon source has similar prebiotic activity as the inulin carbon source and has a stronger effect on *L. casei* than inulin.

As shown in Figure 9D, the 72 h growth curve of 60%-LSP-UMSE carbon source culture of *L. casei*, the delay period is 0–4 h, the growth period is 4–24 h, where 4–18 h is the logarithmic growth period, and the growth rate of bacteria concentration from 18 to 24 h decreases rapidly to 0 to enter the stable period, with linear dependence in the concentration range of 0–2.0%. The growth rate of *L. casei* increased with increasing concentration and entered the stabilization period at least 4 h earlier without decay. In general, the 60%-LSP-UMSE carbon source increased the growth rate of the logarithmic growth period compared to the inulin carbon source, and allowed it to enter the stabilization period earlier.

As shown in Figure 9E, the 72 h growth curve of *L. casei* with 80%-LSP-UMSE carbon source showed that 0–4 h was in the delay period, 4–24 h was in the growth period, where 4–20 h was the logarithmic growth period, and 20–28 h showed a rapid decrease in growth rate to 0 and entered the stable period. The 80%-LSP-UMSE carbon source showed a linear correlation between carbon source concentration and bacterial concentration in the range of 0–2.0%. Overall, the 80%-LSP-UMSE carbon source showed probiotic activity against *L. casei* and increased the growth rate, and reached maximum growth earlier than the inulin carbon source during the logarithmic growth period.

As shown in Figure 9F, the acidity of the fermentation broth was greater than that of inulin in the range of 0.5–2.0% when LSP was used as the carbon source and showed significant differences (*p < 0.05*), and the overall acidity value in this concentration range showed a non-linear increase with increasing concentration. Among them, 40%-LSP-UMSE and 80%-LSP-UMSE had similar effects on acid production of *L. casei*, and the order of the four effects on acidity of *L. casei* culture was 40%-LSP-UMSE ≈ 80%-LSP-UMSE >60%-LSP-UMSE > inulin, indicating that LPS is more productive and value-added than inulin when used as a carbon source.

#### 3.6.5. Probiotic effects of polysaccharides carbon source on *Lactobacillus casei*

**Figure 10:**
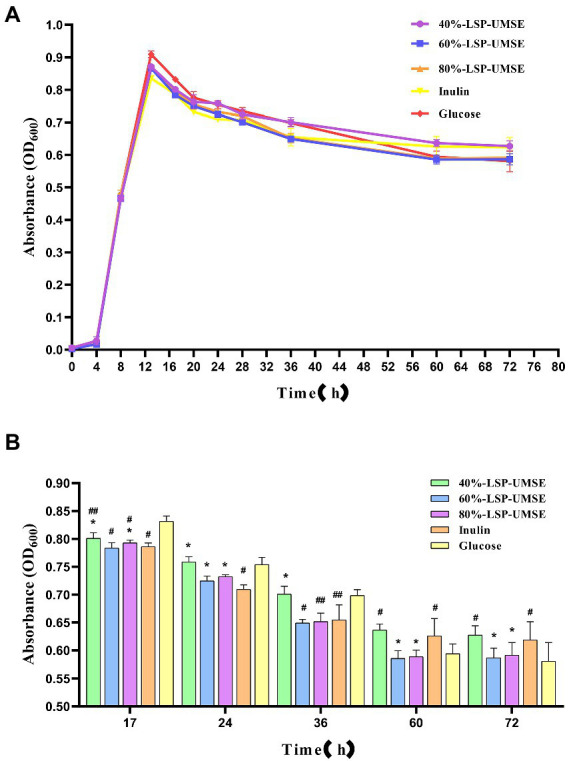
**(A)** Growth promotion curve of 0.5% complex polysaccharide (monosaccharide: polysaccharides = 9:1) MRS; **(B)** OD_600_ of 0.5% complex polysaccharide (monosaccharide: polysaccharide = 9:1) MRS culture. (Note: inulin was used as control, * was significantly *p* < 0.05; when glucose was used as control, # was significantly *p* < 0.05.)

From the results in Figure 9, it can be concluded that LSP as a carbon source has stronger prebiotic activity than inulin for *L. casei* and relatively prolongs the logarithmic growth period and extends the stability period, so the subsequent experiment was completed with a monosaccharide-polysaccharide ratio of 9:1 in place of partial glucose. As shown in Figure 10, when incubated to 14 h, the OD_600_ of *L. casei* cultured with 40%-LSP-UMSE, 60%-LSP-UMSE, 80%-LSP-UMSE and inulin MRS were all less than 0.5% glucose MRS, i.e., no significant value-added effect was reflected, while 40%-LSP-UMSE and 80%-LSP-UMSE were higher than OD_600_ of inulin MRS as carbon source culture and there was a significant difference, i.e., *L. casei* utilized LSP more than inulin, which is consistent with the results of the above data. During the decay period from 14 to 36 h, there was a decreasing trend in OD_600_ of the four polysaccharides MRS and 0.5% glucose MRS cultures of 40%-LSP-UMSE, 60%-LSP-UMSE, 80%-LSP-UMSE and inulin, but the OD_600_ of LSP was significantly higher than that of the carbon source of the inulin group, while it was not significantly different from that of the 0.5% glucose group significant difference and showed no decay-slowing effect. During the decay period from 36 to 60 h, the OD_600_ of the four polysaccharides MRS was significantly smaller than that of the 0.5% glucose MRS, and the OD_600_ of the 40%-LSP-UMSE and inulin groups was significantly higher than that of the glucose group at 60 h. After 60 h, the decay rate was close to 0, and the growth curve had tended to a stable phase, and the OD_600_ of the 60%-LSP-UMSE and 80%-LSP-UMSE were not significantly different from that of 0.5% glucose group but lower than that of inulin MRS. The results showed that LSP under the growth curve of the complex polysaccharide medium with this degree of substitution all slowed down the decay of *L. casei* after reaching the maximum growth with 0.5% glucose carbon source to some extent, but both 40%-LSP-UMSE and inulin increased the final growth.

**Figure 11:**
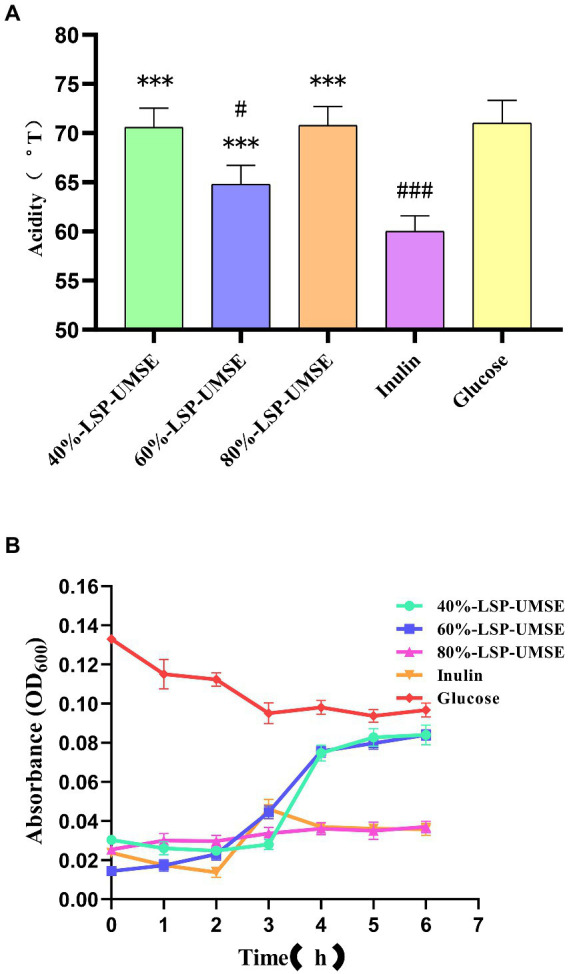
**(A)** Comparison of acidity of 0.5% polysaccharides (glucose: polysaccharide =9:1) MRS medium at 24 h. **(B)** Bile salt tolerance of *L. casei* cultured with different carbon sources. (Note: inulin was used as control, *** was extremely significantly *p* < 0.005; when glucose was used as control, # was significantly *p* < 0.05; ### is extremely significant, *p* < 0.005).

During the proliferation of lactic acid bacteria-like bacteria will produce a large amount of acidic metabolites, they produce the main acidic end products such as lactic acid and acetic acid through homotypic and heterotypic fermentation, and some studies have shown that the acidic metabolites produced by lactic acid bacteria fermentation have a strong function of inhibiting harmful bacteria. Acid production leads to an increase in the acidity value of the fermentation broth, and as the culture time grows, the bacterium produces more and more acid and the pH of the fermentation broth decreases. The effect of LPS addition on the change of acidity in *L. casei* fermentation broth is shown in Figure 11A. The acid production capacity of *L. casei* in the fermentation broth with LPS addition was higher than that of inulin at all stages of fermentation, and there was no significant difference between the acidity values of 40%-LSP-UMSE and 80%-LSP-UMSE and glucose. Significant difference, indicating that the addition of LPS could improve the acid production capacity of *L. casei* and the acid production was improved.

#### 3.6.6. Determination of bile salt tolerance activity

As shown in Figure 11B, *L. casei* could be grown in 40%-LSP-UMSE, 60%-LSP-UMSE, 80%-LSP-UMSE, and inulin media containing 0.3% bile salt concentration, while the OD_600_ of *L. casei* showed a significant decrease in the carbon source of glucose, with 40%-LSP-UMSE and 60%-LSP-UMSE as the carbon source after culturing *L. casei* significantly increased its bile salt tolerance activity relative to inulin and glucose. *L. casei* cultured with 80%-LSP-UMSE and inulin as carbon sources did not show a trend of decreasing bacterial concentration compared to glucose.

The results of the growth curve and acidity histogram showed that the prebiotic activity of 40%-LSP-UMSE and 80%-LSP-UMSE was significantly better than that of inulin; the 0.5% complex polysaccharide MRS with a monosaccharide-polysaccharide ratio of 9:1 did not show growth-promoting activity, and 40%-LSP-UMSE had a better probiotic effect in carbon source verification than inulin in terms of slowing down decay and increasing final OD_600_ and acid production. Improved final OD_600_ and acid production, while 40%-LSP-UMSE and 80%-LSP-UMSE showed better acid-producing prebiotic effects compared to inulin in the carbon source validation. For the study of bile salt tolerance activity, it was found that LPS as a carbon source to culture *L. casei* increased its bile salt tolerance activity compared to inulin and glucose.

## 4. Discussion

Fungal polysaccharides as one of the main active ingredients of medicinal fungi, after years of research development, scientific researchers found that various polysaccharides of fungi such as Poria and Shiitake have a variety of physiological activities such as anti-tumor, anti-inflammatory, immune modulation, anti-mutagenesis, anti-virus, lipid regulation and other effects (Qiang et al., 2012; Zhong et al., 2013; Miao et al., 2013b; Fujia et al., 2017; Mohd et al., 2017; Liuming et al., 2020; Sun et al., 2020, 2022; Tan et al., 2020; Goldman et al., 2021; Xu et al., 2021). In addition to the above biological activities, it was also found that fungal polysaccharides can induce the production of NGF (Nerve growth factor) in nerve cells (Lai et al., 2013), and have a significant improvement effect on the symptoms of cognitive impairment (Kazutoyo, 2012). Fungal polysaccharides also have significant benefits on the cardiovascular system, can significantly reduce blood glucose concentration cholesterol, and have a good role in regulating blood glucose (Jinn et al., 2005; Tan et al., 2020), and its health care value and medicinal value are of great concern. Polysaccharides have been used clinically to treat certain diseases; fungal polysaccharide products such as shiitake mushroom polysaccharides and yunzhi polysaccharides are already available in the market, further confirming the attractive development prospect of fungal polysaccharides. *L. sordida* is a kind of edible mushroom with delicious taste and high nutritional value, and the literature reports that its nutritional composition is rich and diverse and has a variety of biological activities, and the biological activities that have been reported so far include antioxidant, anti-aging, antibacterial, and immune enhancement, etc. The pharmacological activities of the LSP are being developed gradually, and its economic and medicinal values are becoming more and more prominent.

A series of reactive oxygen species, such as hydroxyl radicals, superoxide radicals and hydrogen peroxide, are produced during cell metabolism and growth, and these reactive oxygen species can damage proteins and nucleic acids in the body, leading to disease, and antioxidation is an important way to address the excess of free radicals, so the study or search for substances that exhibit antioxidant functions to reduce or eliminate the presence of free radicals is a current hot topic of research in various fields (Jingjing et al., 2012; Zhong et al., 2013). Some studies have reported significant antioxidant activity efficacy of *L. sordida* (Zhong et al., 2013), which acts mainly as an LSP. Zhong et al. (2013) extracted intracellular polysaccharide (CLSP) from *L. sordida* mycelium and found that CLSP had significant scavenging activity against superoxide anion, hydroxyl radical and DPPH radical, and CLSP significantly inhibited malondialdehyde (MDA) formation in brain and serum of mice and increased superoxide dismutase (SOD) and glutathione peroxidase (GSH-Px) activity in a dose-dependent manner. Xu et al. (2021) investigated the antioxidant effect of *L. sordida* mycelia polysaccharide (LSAP) by from and found that LSAP had significant effects on DPPH radical scavenging, hydrogen peroxide scavenging, lipid peroxidation capacity, reducing capacity and Fe* chelating properties. These are consistent with the results of the present study. In this paper, we found that LPS-UMSE had stronger scavenging ability for ABTS radicals, DPPH radicals, hydroxyl radicals and iron reduction compared to LSP-HWE by antioxidant analysis, indicating that LPS had stronger antioxidant activity.

Recent studies have found that probiotics not only regulate intestinal microbial balance (Li et al., 2018; Kumar et al., 2022), but are also relevant to diseases that are harmful to human health, such as inflammation (Yilmaz et al., 2022), tumors (Mohd et al., 2022), and leukemia (Fric, 2007). The micro-ecological imbalance of intestinal flora should not be ignored. Probiotics can also treat many diseases when used appropriately. The possible mechanisms by which probiotics can effectively alleviate autoimmune diseases are correcting intestinal flora imbalance, increasing the proportion of probiotic flora, promoting intestinal wall and mucosal repair, inhibiting bacterial proliferation and their metabolites translocation, and suppressing the production of pro-inflammatory factors (Sun et al., 2020). The use of probiotics in combination with triple therapy is effective in treating patients with H. pylori-associated chronic gastritis, which not only effectively improves the inflammatory response of patients, but also reduces the occurrence of adverse reactions with a high safety profile (Lu et al., 2022). When probiotics are used together with antibiotics, they can significantly reduce the risk of Antibiotic related enteritis (AAD) in patients, and can also slow down the clinical symptoms of AAD (Singhi and Baranwal, 2008). Studies have shown that when pathogenic and beneficial bacteria grow together and use prebiotics, their population balance changes to some extent, which favors the beneficial bacteria (Jung et al., 2015). We believe that this may be because beneficial bacteria, such as Bifidobacterium, Lactobacillus casei, etc., lower the pH level by producing acid when using prebiotics, making the intestine favorable for pathogen growth (Pereira et al., 2020). In addition, because of the acid production by lactic acid bacteria and bifidobacteria using prebiotics, the production of SCFA, IL-10, etc. increases thereby inhibiting the proliferation of harmful bacteria (Angela et al., 2020). Probiotics as one of the current research hotspots, and the balance of intestinal flora microecology is closely related to every person’s health, fungal polysaccharides have prebiotic activity, which can promote the growth and proliferation of probiotics and acid production and other effects. The prebiotic activity of LSP is less studied at present. Today, probiotic preparations are slowly becoming common, and the role of probiotics on human health is accepted and recognized by the public.

In this paper, we analyzed the effect of three graded alcoholic polysaccharides fractions on the proliferation of *L. casei* using parthenogenic anaerobic microenvironment, *in vitro* fermentation culture method, and the effect of LSP on the growth, acid production and activity of *L. casei* using low doses of added polysaccharides. We can further choose to add polysaccharides directly based on MRS medium or compound polysaccharides to investigate the effect of probiotic activity; regarding the study of probiotic tolerance, this thesis obtained the improvement of bacterial activity after polysaccharides culture through bile salt tolerance experiment, and we can study the effect of LSP on the gastrointestinal stability and acid tolerance of probiotics in the future.

## 5. Conclusion

We obtained LSP from *L. sordida* mycelia using the UMSE method. Based on their chemical properties and antioxidant activity, by comparing the growth, proliferation and acid production effects of Lactobacillus casei. The results showed that all three LPS-UMSE cultures as carbon sources were able to increase the acidity value of *L. casei* and improve the biomass of the bacteria, and we found that the probiotic activity of the three polysaccharide fractions was significantly better than that of inulin. These three polysaccharides significantly increased the bile salt tolerance activity of *L. casei* when used as a carbon source compared to glucose. LPS as a potential prebiotic and intestinal immune enhancer is a natural health-enhancing functional food ingredient. This study is also important for broadening the development of polysaccharide prebiotics in biological functions and related products.

## Data availability statement

The original contributions presented in the study are included in the article/supplementary material, further inquiries can be directed to the corresponding author.

## Author contributions

LaW, QZ, JL, and DY: formal analysis. LaW, JL, YW, QZ, and DY: funding acquisition and investigation. LaW, JL, QZ, LiW, YW, and DY: methodology. LaW, QZ, JL, and YW: project administration. LaW and LiW: resources. LaW: software. DY: supervision. LaW: writing – original draft. All authors contributed to the article and approved the submitted version.

## Funding

This research was supported by grants from the Key scientific research projects of general universities in Guangdong Province (2019KTSCX222, 2019KTSCX221, and 2019KQNCX200) and Zhuhai College of Science Technology “2021 Doctoral promotion program” to LaW.

## Conflict of interest

The authors declare that the research was conducted in the absence of any commercial or financial relationships that could be construed as a potential conflict of interest.

## Publisher’s note

All claims expressed in this article are solely those of the authors and do not necessarily represent those of their affiliated organizations, or those of the publisher, the editors and the reviewers. Any product that may be evaluated in this article, or claim that may be made by its manufacturer, is not guaranteed or endorsed by the publisher.
